# HIV prevalence in the Israeli tuberculosis cohort, 1999–2011

**DOI:** 10.1186/1471-2458-14-1090

**Published:** 2014-10-21

**Authors:** Mor Zohar, Lidji Moshe, Chemtob Daniel, Cedar Noa, Grotto Itamar

**Affiliations:** Department of Tuberculosis and AIDS, Ministry of Health, Jerusalem, Israel; Ramla Department of Public Health, Ministry of Health, 3 Danny Mass St, Ramla, 72100 Israel; Tel-Aviv Tuberculosis Clinic, League against Tuberculosis and Lung Diseases, Tel Aviv, Israel; Public Health Services, Ministry of Health, Jerusalem, Israel; Faculty of Medicine, Ben Gurion University in the Negev, Beer Sheva, Israel

**Keywords:** AIDS, Co-infection, Extra-pulmonary tuberculosis, HIV, Israel, Tuberculosis

## Abstract

**Background:**

Tuberculosis (TB) patients who are co-infected with HIV are at greater risk of mortality. Nevertheless, not all countries achieved sustainable and TB and HIV collaboration to describe the burden of both diseases at a national scale. This study aims to describe HIV prevalence among TB-patients in Israel and identify variable associated with TB/HIV co-infection.

**Methods:**

This retrospective study was conducted by cross-matching the National HIV and TB Registries to describe TB/HIV epidemiology during the last 13-years and define variables predicting TB/HIV co-infection.

**Results:**

Between 1999 and 2011, 5,502 TB-patients were reported: 779 (14.2%) were Israeli-born and 4,723 (85.8%) non-Israeli born. Of all TB patients, 254 (4.6%) were HIV-infected. The trend of HIV/TB co-infection among non-Israeli born has generally decreased since 2003 (trend analysis *p* < 0.001).

TB/HIV co-infected patients were mostly males, their TB diagnosis had been performed relatively in shorter time following their arrival in Israel, more likely to be in the 35–44 and 25–34 age groups, non-Israeli born (mostly Africa born), more likely to be culture positive, have multi-drug resistant strains, had worse treatment outcomes and more likely to die treatment than HIV-negative tuberculosis patient. In a multivariate analysis, short time after arrival in Israel, older age, being born in Ethiopia, having positive sputum, positive culture and multi-drug resistant TB predicted TB/HIV co-infection.

TB/HIV co-infected patients with extra-pulmonary TB had a higher proportion of infection in lymphatic, miliary and abdominal sites than those with extra-pulmonary TB who were HIV-negative.

**Conclusions:**

Most TB/HIV co-infected patients were migrants originating in high-burden countries. Despite the moderate 4.6% TB/HIV co-infection rate in Israel, these patients had worse treatment outcomes and higher mortality rates. This study illustrates the importance of integrating TB with HIV in surveillance and treatment components, which should be employed in other countries, as it has a positive impact on disease control.

## Background

One third of the world's population (2.2 billion people) is infected with *Mycobacterium tuberculosis,* and about 9 million of those develop tuberculosis (TB) disease annually [1]. TB also caused more than 1.4 million deaths globally in 2009, which equals to approximately 160 deaths per hour [2]. Entering the fourth decade of the AIDS epidemic, it is estimated that there are 33.3 million individuals who are living with HIV/AIDS, mostly in scare resources countries [3], located squarely in similar regions affected by HIV. The impaired CD4 cells in HIV-infected individuals, which are the preferred habitat of *Mycobacterium tuberculosis*, are not able to activate macrophages. The life time risk that a person with TB infection will develop active TB disease is 5-10%, while the risk is 21–34 times higher in people living with HIV [1]. TB/HIV co-infection is deadly, and of the 1.1 million persons infected with both TB and HIV in 2009, 0.35 million died, although preventive measures are widely available and effective therapy is provided for free for both diseases in most parts of the world.

Diagnosing TB among HIV-infected patients is challenging, as they may have negative sputum-smears and up to one-third may show normal chest-radiographs, thus may evade routine diagnostic procedures. Additionally, extra-pulmonary TB is more common in HIV-infected patients, and may be undiagnosed until later stages of the disease [2].

Both TB and HIV are prevalent in most industrialized countries among migrants originating from high HIV- or TB-burden countries, and also among intravenous drug users (IVDU) [4]. There are strategic frameworks to reduce the burden of TB and HIV outlining the need for political commitment, collaborative prevention, intensified case-finding, coordinated treatment and strengthening surveillance [5]. Early detection of each of the diseases and treatment initiation is the backbone of prevention strategies. Effective treatment which is provided in earlier stages decreases mortality and reduces further transmission in the community. Nevertheless, not all countries achieved sustainable TB and HIV collaboration, and their surveillance systems should be improved [6].

This retrospective study describes HIV-prevalence among TB patients notified in Israel between 1999 and 2011, and identifies demographic, clinical and laboratory characteristics of co-infected patients.

## Methods

This observational cohort study was based on the National TB and HIV Registries. Active TB disease, suspected TB infections, HIV and AIDS are reportable conditions in Israel by law. Each notification sent to the Ministry of Health includes demographic, clinical, laboratory and treatment information.

A TB case was defined as culturally confirmed disease due to *Mycobacterium tuberculosis* complex from sputum, body fluids or tissue; or clinician's judgment to treat a patient with a full course of anti-tuberculosis treatment when the patient's clinical or radiological signs and/or symptoms were compatible with TB. Treatment outcomes were defined by the WHO [7], and patients who were cured or completed the treatment were considered success. TB diagnostic procedures and treatment for tuberculosis are fully covered by the various health insurers for Israeli citizens and reimbursed by the Ministry of Health in case of undocumented migrant workers. TB treatment is provided by directly observed therapy for the entire treatment period.

HIV tests are recommended for all TB patients in Israel, and the average estimated HIV testing coverage during the study period was 88.4% [8]. HIV tests in Israel are confidential, provided at no cost and are offered universally. The diagnosis of HIV/AIDS in Israel requires two positive enzyme-linked immunosorbent assay results and a confirmation by the western blot blood test.

Populations at high-risk for developing TB and/or HIV are screened routinely. Jewish Ethiopians migrants are screened for TB in Ethiopia and for HIV upon arrival while being naturalized. Documented migrant workers are screened for TB and HIV in their countries of origin before being granted a work visa. Undocumented migrant who are detained in jail for entering the country without a valid visa are screened for TB before they are discharged to the community. IVDU are screened for both diseases before undertaking rehabilitation programs.

The National TB and HIV Registries are matched periodically, as both diseases are reported nominally to the same department at the central level. As this was routine epidemiological analysis on the non-identified data within a standard public health database reporting system, ethics approval was not required [9].

All TB patients who were reported in Israel were followed from 1 January 1999 and until 31 December 2011. Trend analysis was performed by the chi-square to yield the linear-by-linear association test. Categorical and continuous variables were compared between TB-patients who were co-infected with HIV and TB-patients who were HIV-negative by the chi-square and Students-*t* test, respectively. *P* < 0.05 was considered statistically significant. Univariate analysis of demographic, clinical and laboratory characteristics associated with TB/HIV co-infection was performed, generating odds ratios (OR) and 95% confidence intervals (CI). Dependent variables achieving statistical significance in the univariate analysis were included in the multivariate analysis predicting TB/HIV co-infection. All analyses were performed by the SPSS® program 18.0 (Statistical Package for the Windows, College Station, TX, USA).

## Results

**Figure 1:**
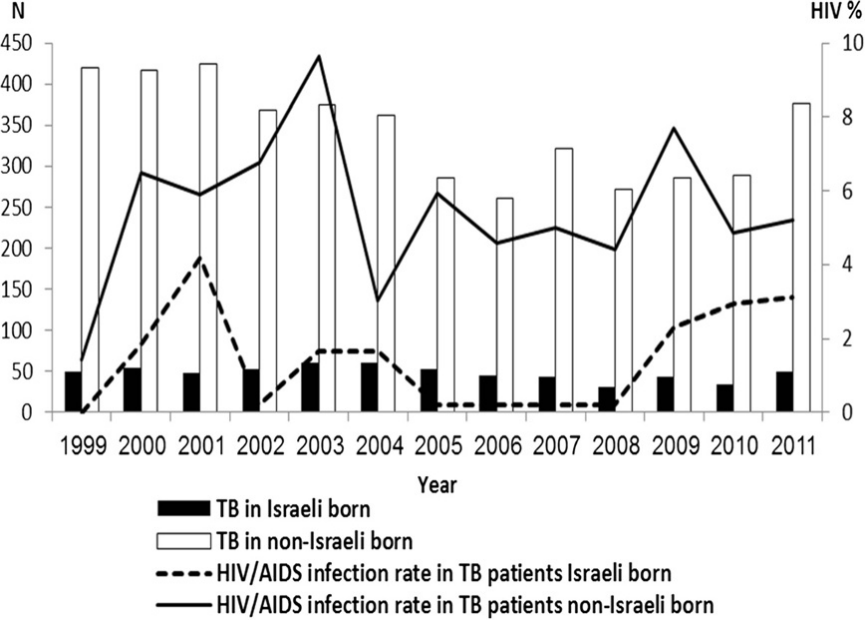
**HIV infections of tuberculosis patients in Israel, by country of birth, 1999–2011.** Tuberculosis incidence among Israeli-born between the years 1999 to 2011 was low yet sable, while the incidence of tuberculosis in non-Israeli born was 7–10 times higher. The rate of tuberculosis incidence among non-Israeli born has increased from 2006 and onward. The rate of HIV in Israeli born tuberculosis patients was relatively low, but increased from 2008 and onward, while the rate of HIV in non-Israeli born tuberculosis patients was 2–3 times higher and generally stable.

Between 1999 and 2011, 5,502 TB patients were reported to the Ministry of Health in Israel. Of those, 779 (14.2%) were Israeli born and 4,723 (85.8%) non-Israeli born. Of all TB patients diagnosed in Israel and tested for HIV, 254 (4.6%) were co-infected. The trend of TB/HIV co-infection among non-Israeli born has generally decreased since 2003; while among Israeli-born, the trend has been stable in last years (trend analysis *p* < 0.001, Figure 1). Although the small number of Israeli-born who were co-infected during the last three years (N = 6), four were IVDU and the other two were infected from a household member. In comparison to TB patient who were HIV-negative, those who were TB/HIV co-infected were mostly males, their TB diagnosis was performed relatively in a short time following their arrival in Israel, more likely to be 35 years or older, non-Israeli born (mostly born in Ethiopia), more likely to be culture positive and have multi-drug resistant strain, had worse treatment outcomes and more likely to die during treatment (Table 1). In a multivariate analysis, short time after arrival in Israel, older age, being born in Ethiopia, having positive sputum positive culture and multi-drug resistant TB predicted TB/HIV co-infection.

**Table 1:** **Tuberculosis patients in Israel, by HIV-infection status and co-infection risk, 1999-2011**

Characteristic		TB patients co-infected with HIV N = 253 (%)	TB patients without HIV N = 4797 (%)	***P***	Univariate OR (95% CI)	Multivariate OR (95% CI)
Males		165 (65.0)	2837 (59.1)	0.04	1.2 (1.1-1.3	1.5 (0.6-3.8)
Time from arrival in Israel to TB diagnosis. Median years (range)		5.5 (0–32)	6.0 (0–83)	0.001	0.8 (0.7-0.9)	0.8 (0.7-0.9)
Age group at TB diagnosis in years	18-24	13 (5.1)	453 (9.4)	0.01	0.2 (0.08-0.5)	0.4 (0.2-1.1)
	25-34	85 (33.5)	1041 (21.7)		0.07 (0.03-0.1)	0.1 (0.05-0.3)
	35-44	87 (34.3)	763 (15.9)		0.05 (0.02-0.1)	008 (0.04-0.2)
	45-54	45 (17.7)	604 (12.6)		0.7 (0.03-1.2)	0.8 (0.1-1.3)
	55-64	15 (5.9)	510 (10.6)		5.2 (2.3-12.2)	3.2 (2.1-8.6)
	>65	8 (3.1)	1436 (29.8)		1	
Israeli citizens		198 (78.3)	3886 (81.0)	<0.001	1.2 (0.9-1.6)	
Country/area of birth	Israel	11 (4.3)	604 (12.6)	<0.001	1	
	Ethiopia	137 (54.1)	858 (17.9)		4.6 (1.4-15.3)	6.5 (1.6-25.5)
	Horn of Africa	15 (5.9)	192 (4.0)		0.3 (0.1-0.6)	0.8 (0.6-1.7)
	Former Soviet Union	50 (19.8)	1629 (34.0)		0.3 (0.1-0.8)	0.3 (0.2-0.6)
	Europe	4 (1.6)	320 (6.7)		1.7 (0.5-6.4)	1.9 (0.8-2.5)
	South east Asia	6 (2.4)	461 (9.6)		1.2 (0.5-3.5)	1.1 (0.3-3.3)
	Others	31 (12.2)	733 (15.3)		1.8 (0.7-2.1)	1.3 (0.9-1.7)
Positive culture		211 (83.4)	3612 (75.3)	<0.001	1.5 (1.12-2.2)	1.9 (1.3-3.0)
Multi drug resistance		19 (7.5)	189 (3.9)	0.007	1.9 (1.2-3.1)	1.9 (1.1-3.3)
Pulmonary TB		203 (80.2)	3876 (80.8)	0.7	1.1 (0.7-1.4)	
Treatment success*		179 (70.8)	3953 (82.4)	<0.001		
Died during treatment		43 (17.0)	492 (10.3)	<0.001		

*Completed treatment or cured, as defined by the WHO.

CI = Confidence interval.

OR = Odds ratio.

TB = Tuberculosis.

The proportion of extra-pulmonary TB of all TB patients was not statistically different between TB/HIV co-infected and those who were not HIV-infected, *p* = 0.7. However, TB/HIV co-infected patients had a higher proportion of lymphatic, miliary and abdominal TB than those who were HIV-negative (9.8% *vs*. 7.6%, 2.0% *vs*. 0.2% and 2.8% *vs*. 1.4%, respectively, *p* < 0.01).

## Discussion

In this nationwide retrospective cohort study, it was found that during the 13 years of follow-up, an average 4.6% of all TB patients were co-infected with HIV, in decreasing in rate since 2003. Most TB/HIV co-infected patients were migrants older than 35 years of age, originating in high TB and HIV prevalence countries and having multi-drug resistant TB-strain.

The co-infection rate of 4.6% found in our study was slightly lower than that reported from the ECDC [8], CDC [10], Canada and Australia [11], which published 5.5%, 6%, 6% and 5%, respectively. TB/HIV co-infection rates in Israel were also lower than western European countries [12]. Most TB/HIV co-infected patients in this study were migrants, especially originating in Africa, as 34,127 Jewish Ethiopian legal migrants and 37,646 illegal migrants from the horn of Africa (Eritrea, Sudan and Ethiopia) arrived in Israel after 2000. It is probable that the vigilant screening procedures of migrants from high TB- and HIV-prevalence countries which are performed in Israel detected TB in initial its stages, as well as latent TB infections, allowing early treatment initiation [13]. Israel has established screening programs for legal Ethiopian migrants [14] and other undocumented migrants from the horn of Africa [15], to allow for early diagnosis and treatment in among migrants originating in countries characterized by high TB/HIV prevalence.

TB diagnosis is more complicated among HIV/AIDS-infected patients, as the clinical presentation of TB might be atypical. In order to prevent death among TB/HIV co-infected patients, modified anti-retroviral treatment (ART) and isoniazid preventive therapy (IPT) should be prescribed. It was recently published that the patients should be treated with ART 2 weeks after initiation of TB-treatment, rather than sequential treatment regimen by starting ART only as TB treatment had been completed [16]. These recommendations were established despite the possible overlap ART toxicity profile, which may cause immune reconstitution inflammatory syndrome (IRIS), while the recovering immune system regains its regulatory function.

The rate of extra-pulmonary TB in our study was not greater in TB/HIV co-infected patients than in HIV-negative TB patients, as also found by other studies, however, their prognosis was worse [17]. It is possible that the lack of association between extra-pulmonary TB and HIV in this study was a result of analyzing only extra-pulmonary TB cases, rather than concurrent pulmonary and extra-pulmonary TB in HIV patients, as defined by the WHO, resulting in a possible reporting bias. The higher rates of lymphatic, miliary and abdominal TB were in agreement with other studies, as immunodeficiency probably contributed to the development of more severe forms of TB [18].

TB/HIV co-infected patients are more susceptible to multi-drug resistant TB strains, either through nosocomial transmission, as they visit medical settings more commonly; or due to malabsorption of TB-medication, as the ART may cause rapid gastrointestinal passage; or by poor response to TB treatment as a consequence of other social difficulties, such as being a migrant or drug-user [18]. This finding underscores the importance of prompt TB diagnosis and early treatment in HIV-infected patients in order to prevent drug resistance developing and further *Mycobacteria* spread. Screening program of migrants is effective in early detection, especially as most of the TB/HIV co-infected migrants developed TB within the first two years following their arrival in Israel [19].

As higher rate of patients who were co-infected with TB/HIV died during treatment than TB patients who were HIV-negative, and because HIV presents a significant challenge to TB control, effective efforts are crucial in maintaining TB/HIV collaborative activities, such as an accurate monitoring and evaluation of the performance of the National TB program. The "3 Is": (intensified case finding, isoniazid prophylaxis and improved infectious control) [20] should be incorporated in the core services for prevention and treatment of HIV infection. The "3 Is" strategy should be augmented with ART early following TB diagnosis in order to support restoration the immune system [16].

In most developed countries, TB/HIV co-infection burden is unknown, as data confidentiality precludes the notification of HIV status of TB patients [6]. Consequently, those countries are unable to cross-match the data due to regulations respecting patients' confidentiality, or that data are registered in different agencies, and other technical obstacles, such as difficulties to spell correctly the names of the migrants. Israel is an exception in these situations, as both National TB and HIV Registries are based on patients' names and identification numbers, and are managed by the same department, enabling a periodical identified cross-matching, ensuring completeness of reporting and reducing timeliness. Cross-matching allows the Israeli Ministry of Health to identify populations at risk, thus appropriating interventions aiming to decrease co-infection, and enabling central evaluation of the performances of both TB and HIV/AIDS clinics to detect and treat HIV and TB in their patients.

This study is subject to several limitations that may merit discussion. First, there were no accurate data regarding the proportion of TB patients who were tested for HIV. Nevertheless, high-risk groups for developing TB or HIV in Israel are routinely screened, and also financial or administrative incentives are granted to the treating centers. We reported estimation greater than 85% coverage of HIV-testing among TB, which was higher than the set benchmark call of the WHO for testing and counseling [21]. Second, our data-base did not include behavioral determinants of those infected with HIV. However, most of the migrants in israel were mainly infected by heterosexual contacts [22].

## Conclusion

In conclusion, this uniform long term follow-up showed that 4.6% of all TB patients were also infected with HIV. Most of them were migrants older than 35 years of age, originating in high-burden countries and having multi-drug resistant TB-strain. Despite the relatively moderate TB/HIV co-infection rate in Israel, co-infected patients had worse treatment outcomes and are more likely to die during treatment. Integrating TB with HIV in surveillance and treatment facets is paramount, and improves the quality of disease control.

## Abbreviations

ART: Anti retroviral therapy
HIV: Human immunodeficiency syndrome
IPT: Isoniazid preventive therapy
IRIS: Immune reconstitution inflammatory syndrome
IVDU: Intravenous drug users
TB: Tuberculosis.
