# Erratum to: ‘HIV prevalence in the Israeli tuberculosis cohort, 1999–2011’

**DOI:** 10.1186/s12889-016-2889-0

**Published:** 2016-04-13

**Authors:** Zohar Mor, Moshe Lidji, Daniel Chemtob, Noa Cedar, Itamar Grotto

**Affiliations:** Department of Tuberculosis and AIDS, Ministry of Health, Jerusalem, Israel; Ramla Department of Public Health, Ministry of Health, 3 Danny Mass St, Ramla, 72100 Israel; Tel-Aviv Tuberculosis Clinic, League against Tuberculosis and Lung Diseases, Tel Aviv, Israel; Public Health Services, Ministry of Health, Jerusalem, Israel; Faculty of Medicine, Ben Gurion University in the Negev, Beer Sheva, Israel

Unfortunately, the original version of this article [1] contained an error in author names. The correct names are listed below (first name and last name) and also corrected above:

- Zohar Mor
- Moshe Lidji
- Daniel Chemtob
- Noa Cedar
- Itamar Grotto
